# Corelation between Machines Assisted Endodontic Irrigant Agitation and Apical Extrusion of Debris and Irrigant: A Laboratory Study

**DOI:** 10.1155/2014/346184

**Published:** 2014-10-16

**Authors:** Jatin Gupta, Vineeta Nikhil, Padmanabh Jha

**Affiliations:** Department of Conservative Dentistry and Endodontics, Subharti Dental College, NH-58 Delhi-Haridwar Road, Meerut, Uttar Pradesh 250005, India

## Abstract

*Aims.* To compare amount of root canal debris and irrigant extruded apically after irrigants agitation using closed and open chambers. *Methods and Material.* Sixty maxillary central incisors were selected, decoronated, and mounted in preweighed glass vials filled with distilled water. Biomechanical preparation was completed using ProTaper rotary files until number F4 and 1 mL of 3% NaOCl solution after each file use. Samples were randomly divided into closed or open chamber sets which were further subdivided into 6 groups, based on the agitation techniques: no agitation (control), canalBrush, lentulospiral, passive ultrasonic agitation (PUA), EndoActivator, and EndoVac. Canals were irrigated with 1 mL of 17% EDTA and agitated for 30 s and then flushed with 2 mL of distilled water. Apically extruded irrigant was measured and vials were kept in incubator for 5 days at 68°C for drying for weight calculation. *Statistical Analysis.* Analysis was done using Student's *t*-test, one-way ANOVA, and post-hoc. *Results.* All agitation techniques showed apical extrusion of the debris and irrigant. The closed chamber apparatus showed significantly less extrusion of debris and irrigant than open chamber (*P* < 0.05). *Conclusions.* EndoVac was found to be the safest agitation system among all test groups with regard to apical extrusion of debris and irrigant.

## 1. Introduction

Chemomechanical debridement is an important part of endodontic treatment. Elimination of pulpal tissue, microbiota, and their by-products and removal of organic and inorganic debris by using instruments and endodontic irrigants are objectives of this important phase of treatment [1]. Mechanical debridement either manual or machine assisted fails to clean canal fins, isthmi, cul-de-sacs, and so forth after completion of the preparation and may result in persistent periradicular inflammation [2]. Therefore, irrigation is an essential part of root canal debridement because it allows for cleaning beyond what might be achieved by root canal instrumentation alone [3]. To effectively clean and disinfect the root canal system, an irrigant should be able to disinfect and penetrate dentin and its tubules, offer long-term antibacterial effect (substantivity), remove the smear layer, and be nonantigenic, nontoxic, and noncarcinogenic. Root canal irrigants that are currently used during cleaning and shaping include sodium hypochlorite (NaOCl), chlorhexidine, ethylenediaminetetraacetic acid (EDTA), a mixture of tetracycline, an acid, and a detergent (MTAD). However, there is no one unique irrigant that can meet all the ideal requirements; thus, in contemporary endodontic practice, combinations of irrigants such as sodium hypochlorite (NaOCl) with ethylenediaminetetraacetic acid (EDTA) or chlorhexidine (CHX) are often used to complement the shortcomings that are associated with the use of a single irrigant [3]. Sodium hypochlorite is the most commonly used root canal irrigant and is used in dilutions ranging from 0.5% to 5.25%. Advantages of NaOCl include its ability to dissolve organic substances present in the root canal system and its affordability. The major disadvantages of this irrigant are its cytotoxicity when injected into periradicular tissues, foul smell and taste, and its inability to remove smear layer. Chelating agents such as ethylenediaminetetraacetic acid (EDTA) are used for removal of the inorganic portion of the smear layer. NaOCl is an adjunct solution for removal of the remaining organic components. Irrigation with 17% EDTA for one minute followed by a final rinse with NaOCl is the most commonly recommended method to remove the smear layer [4]. It has been demonstrated that endodontic instrumentation techniques and irrigation protocol tend to cause extrusion of irrigant and debris into the periapical tissues with the consequence of possible postoperative irritation [5]. This extrusion may cause pain, discomfort, and persistent inflammation. Accordingly, any root canal irrigation delivery system that reduces the risk of extrusion into the periapical tissues would greatly benefit patient care [6].

Different agitation techniques have been proposed to improve the efficacy of irrigation solutions, including agitation with irrigation syringe, hand files, gutta-percha cones, canal brush, and sonic and ultrasonic devices [7, 8]. Conventional irrigation with syringes has been advocated as an efficient method of irrigant delivery before the advent of passive ultrasonic activation [3]. The mechanical flushing action created by conventional hand-held syringe needle irrigation is relatively weak. Keir et al. [9] reported improved canal debridement with the use of canal brushes. Significantly improved displacement and exchange of irrigant solution were seen when a well-fitting gutta-percha master cone up was gently moved up and down within an instrumented canal [10, 11]. Tronstad et al. [12] were the first to report the use of a sonic instrument for endodontics in 1985. When compared with sonic irrigation, the more powerful ultrasonic irrigation technique has been shown to be capable of removing more debris [13].

EndoActivator, a sonic frequency agitation system for irrigation, is able to effectively clean debris from lateral canals, remove the smear layer, and dislodge clumps of simulated biofilm within the curved canals of molar teeth [7]. In a preliminary study, Ruddle (2008) [14] has shown that the EndoActivator removes simulated biofilms in extracted teeth. Further, he has shown that hydrodynamics is a function of the canal shape, the size of the activator tip selected, the activation time, the volume of irrigant, the motion of the activator, and the temperature of the irrigant.

The EndoVac system comprises of a macrocannula and microcannula which is connected via tubing to a syringe of irrigant and the high-speed suction of a dental unit. An apical negative pressure irrigation system does not create a positive force at the needles tip so potential accidents can be eliminated. Haas and Edson (2007) [15] found that the teeth irrigated with negative apical pressure had no apical leakage. A study conducted by Fukumoto et al. (2006) [16] concluded that when irrigation devices placed at 2 mm short to working length, apical negative pressure resulted in less extrusion than positive pressure (needle irrigation).

CanalBrush is an endodontic microbrush which is highly flexible and molded entirely from polypropylene. It is designed for removing root canal debris effectively when attached to a contraangle handpiece running at 600 rpm [3]. A study done by Garip et al. (2010) [17] showed that irrigating with canal brush tended to produce cleaner canal walls.

In passive ultrasonic agitation (PUA), the irrigant is activated with the use of an ultrasonically oscillating instrument. The flushing action of irrigants may be enhanced by using ultrasonics. Acoustic streaming has been shown to produce sufficient shear force to dislodge debris in instrumented root canal [18].

Lentulospiral which is commonly used for introducing sealer and pastes in root canal can be expected to agitate the irrigant and may help to push it into lateral canals when used with a slow speed handpiece because of its rotary action. This use as an irrigant agitator has not been explored.

Many of* ex vivo* studies conducted for apical extrusion of debris or irrigant have adopted the model in that each root was attached to an empty collection vial (full of air), where extruded material was collected. Atmospheric pressure in the vial was ensured by communication with the external environment through a large needle which achieved pressure equalization; therefore, a completely open system was employed, without any specific justification. As the apical foramen was surrounded by air, the presence of periapical tissues that could act as a natural barrier and exert some resistance to irrigant extrusion was not simulated [19]. A further modification of the previous model used a vial filled with physiologic saline instead of air to simulate a form of tissue resistance. Nevertheless, an open pathway to the surrounding environment was still ensured by a pressure-equalizing needle, similarly to the original model [20].

This study was designed to compare the apical extrusion of debris and irrigant in open and closed setups when the above-mentioned agitation systems were used in conjunction with endodontic irrigation.

## 2. Materials and Methods

Sixty intact mature permanent maxillary central incisors with single canal and apical foramen and with canal curvature between 0 and 10 degrees were selected and disinfected with 2% thymol solution.

### 2.1. Preparation of Samples

Selected teeth were decoronated at 16 mm length. A number 10 K file was inserted and measured until the tip of the file was just visible at the apical foramen and the working length was established by deducting 1.0 mm from this length. The apical 5 mm of each root tip was covered with cyanoacrylate and a number 20 K-file was protruded 2.5 mm from the apex to create a standard apical constriction of 0.25 mm. The specimens were randomly divided into two groups of 30 each.

*(a) Closed Chamber Set*. Thirty preweighed clean and dry glass vials with rubber stoppers were collected. A hole was created through the centre of each rubber stopper and specimens were inserted under pressure through it up to cementoenamel junction. The margins were sealed with cyanoacrylate. The apical part of the root was suspended within the vial which consisted of premeasured volume (5.5 mL) of distilled water. The apical 3 mm of root tip was submerged in distilled water. There was no direct connection between the root portion submerged in distilled water and atmosphere.

*(b) Open Chamber Set*. Thirty chambers were prepared in the same manner as mentioned above and a bent 27-gauge needle was forced down beside the rubber stopper to balance the air pressure inside and outside. An electronic syringe pump (Uni-Em universal medical instruments, Mumbai, India) was used to deliver the irrigant at a constant flow rate of 0.26 mL/s.

Each group was further divided into 6 equal subgroups (*n* = 5) on the basis of agitation technique used.

### 2.2. Control Group (Closed and Open Sets) (C_NA_/O_NA_)

This group used no form of agitation of irrigant and served as control. Each canal was shaped by crown down technique using the ProTaper rotary system with an endodontic torque control motor (X Smart Dentsply, Maillefer, Ballaigues, Switzerland) until ProTaper size F4. After each file use, irrigation was done with 1 mL of 3% NaOCl solution using Max-i probe tip (30-gauge) (Dentsply International, York, PA). After completion of the preparation, irrigation was done with 1 mL of 17% EDTA for 30 s by Max-I probe needle which was placed 1 mm short of working length. A final irrigation was done with 2 mL of distilled water.

### 2.3. EndoActivator Group (Closed and Open Sets) (C_EA_/O_EA_)

Preparation of canal was done as mentioned previously and after completion of the preparation the canal was filled with 1 mL of 17% EDTA and the EndoActivator (Dentsply/Tulsa Dental Specialties, Tulsa, OK) tip corresponding to number 20 ISO size was inserted 2 mm short of working length and activated for 30 s and finally rinsing was done with 2 mL of distilled water.

### 2.4. Passive Ultrasonic Group (Closed and Open Sets) (C_US_/O_US_)

Preparation of canal was done as mentioned previously and after completion of the preparation, the canal was filled with 1 mL of 17% EDTA, a passive stainless steel ultrasonic file corresponding to number 20 ISO size (IrriSafe, Satelec Acteon, Merignac, France) was kept 1 mm short of working length and was ultrasonically activated by Piezoelectric Ultrasonic unit (DTE, Guangxi China, Mainland) at a power setting of 2 and activated for 30 s, and final rinsing was done with 2 mL of distilled water.

### 2.5. Lentulospiral Group (Closed and Open Sets) (C_LS_/O_LS_)

Preparation of canal was done as mentioned previously and after completion of the preparation, the canal was filled with 1 mL of 17% EDTA and a lentulospiral (Mani, Japan) corresponding to number 25 ISO size was kept 1 mm short of working length and activated at a speed of 300–600 rpm for 30 s. Final rinsing was done with 2 mL of distilled water.

### 2.6. CanalBrush Group (Closed and Open Sets) (C_CB_/O_CB_)

Preparation of canal was done as mentioned previously and after completion of the preparation, the canal was filled with 1 mL of 17% EDTA, a CanalBrush (Coltene/Whaledent GmbH-Co. KG, Langenau, Germany) of medium size corresponding to number 30 ISO size was kept 1 mm short of working length and activated to a maximum speed of 600 rpm in a contraangled handpiece for 30 s, and final rinsing was done with 2 mL of distilled water.

### 2.7. EndoVac Group (Closed and Open Sets) (C_EV_/O_EV_)

Each canal was prepared as described previously. After each file use, irrigation was done with 1 mL of 3% NaOCl solution using EndoVac (Discus Dental, Culver City, CA). Irrigation was done using 17% EDTA with macrocannula which was placed in the coronal and middle third of the canal and microcannula of 25 mm length which was kept up to 0.2 mm short of the working length for 30 s. The final irrigation was done with 2 mL of distilled water.

The volume of the fluid collected in the vial was measured with clean and dry pipettes and micropipettes (US Associates, Lucknow, India). The measured volume of fluid was shifted back to vial and the pipettes were rinsed with 0.5 mL of distilled water to remove any clinged debris from the pipette. Thereafter, the vials were kept in an incubator at 68°C for 5 days until the vials were completely dried.

The vials were then weighed on a precision electronic balance (Shenzhen BOTOO Electronic Technology Co., Ltd., China). The weight of the debris was calculated as the difference between the pre- and postinstrumentation weights of vial. All instrumentation and weighing procedures were carried out by the same operator. Parametric test and SPSS 15 software were used for analysis.

## 3. Results

**Figure 1:**
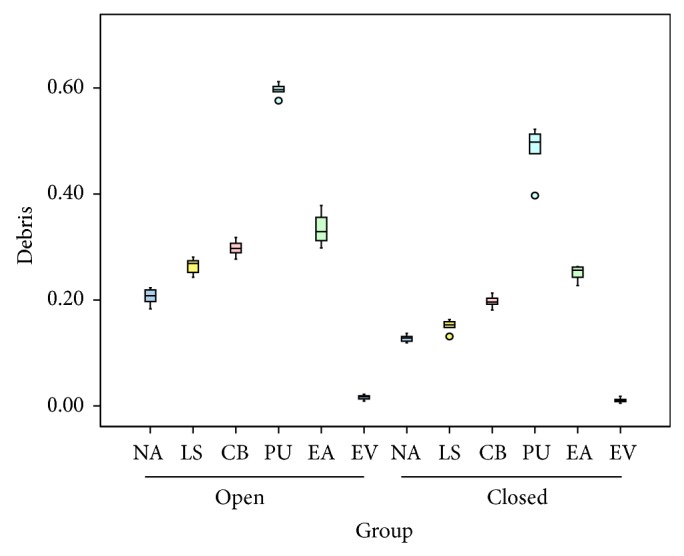
Box diagram showing amount of debris extruded in grams in each groups. Data are presented as mean and standard deviation.

**Figure 2:**
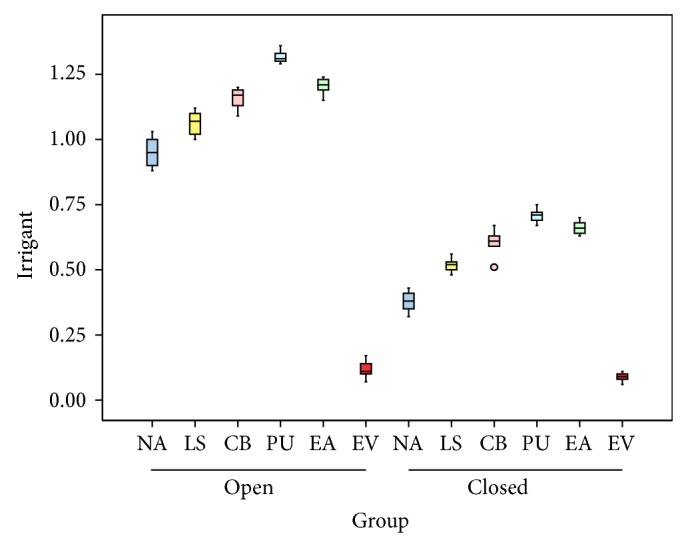
Box diagram showing amount of irrigant extruded in mL in different groups. Data are presented as mean and standard deviation.

The mean and standard deviation was calculated (Table 1) for extruded debris in grams (Figure 1) and extruded irrigant in mL (Figure 2). The statistical analysis was done using Student's *t*-test, one-way ANOVA, and post hoc test (Table 2). All agitation groups except EndoVac and lentulospiral showed significantly more extrusion of debris and irrigant than the controls. Irrespective of the type of chamber, the maximum extrusion of debris as well as irrigant was observed in PUA group while it was minimum for EndoVac group. The difference in case of the lentulospiral was not statistically significant. For all the combinations, open chamber had higher mean apically extruded debris and irrigant values as compared to closed chamber, and the difference between two types of chambers was found to be significant statistically for all the combinations (*P* < 0.05) except EndoVac.

## 4. Discussion

To date no study has been reported on the effect of agitation on apical extrusion. So this study was conducted to quantify the amount of debris and irrigant extruded apically by different agitation systems.

The sectioned samples were mounted on empty glass vials according to the method adopted by Elmsallati et al. [21]. Many previous studies have neglected the effect of periapical resistance by measuring extrusion into vials full of air, which lead to over estimation of irrigant extrusion [21, 22]. In the present model, apical 3 mm of root tip was submerged in distilled water to simulate the resistance offered by the periapical tissues.

The mean amounts of apically extruded debris and irrigant were higher in agitation groups as compared to no agitation except for the EndoVac (*P* < 0.05) and lentulospiral groups. This may be because agitation of fluids in the canal in the experimental group with the mechanically driven instruments created more turbulence of the fluids [23]. EndoVac had the lowest mean values for apically extruded debris and irrigant among all the groups. The reason could be because EndoVac is based on negative apical pressure so during irrigation the negative pressure created by the tip of microcannula lying near the apex siphons off the excess irrigant to prevent overflow [7]. Hargreaves and Cohen also found EndoVac safe to be used to the working length [5].

The values for CanalBrush and lentulospiral for debris and the irrigant were almost equal (*P* > 0.05). This may be because both are based on centrifugal force and were rotated clockwise at the same speed and same distance short of the apex that is 1 mm. As a nontapered instrument has been used in a tapered canal, the tip of lentulospiral might not have been able to move freely in the apical part of the canal, resulting in restricted motions and less of the turbulence in the apical third, causing less extrusion in the present study.

EndoActivator extruded more debris and irrigant (*P* > 0.05) than CanalBrush and lentulospiral but significantly less than PUA in the present study. This might be because the instrument tip was 2 mm short of working length, while in PUA the tip was kept 1 mm short of working length. Additionally, PUA creates cavitation and acoustic microstreaming but sonic energy produces only acoustic microstreaming [24] leading to less removal of debris from the canal walls and which in turn may cause less apical extrusion [25].

The open chamber apparatus had statistically more extrusion (*P* < 0.05) of the debris and irrigant than the closed chamber, except for the EndoVac group (*P* > 0.05). This is in accordance with Psimma et al. [19] who found that pressure equalization (i.e., open chamber) resulted in significantly more extrusion than the sealed vial (i.e., closed chamber), regardless of the vial content due to the absence of resistance offered by periapical tissue against extrusion of irrigant and debris. In open chamber model, there is equalization of atmospheric pressure and pressure inside vial in open chamber apparatus results in a high compliance; thus, there is less resistance offered to the extrusion of irrigant through the apical foramen, and this might represent the clinical condition where the destruction of cortical bone has established a pathway from the apical foramen towards soft tissues, oral cavity, or maxillary sinus [19].

In closed chamber apparatus as the pressure was not equalized, thus the resistance to extrusion of irrigant was maintained. In addition, additional irrigant extrusion occurred through the apical foramen, further resisted the flow of irrigant out of the apical foramen.

The low compliance condition of the closed chamber resembled the clinical condition of a periapical lesion entirely surrounded by cortical bone [19]. Many other studies have tried to simulate the resistance of periapical tissues by different gels [24, 25]. But the use of gels introduced difficulties in quantification of the extruded irrigant [19].

Within the limitation of the study, it can be concluded that all agitation systems result in extrusion of debris and irrigant. Use of EndoVac for irrigation minimizes the extrusion of irrigants and debris while passive agitation with ultrasonics increases the extrusion of both debris and irrigant. Open chamber apparatus method results in more apical extrusion of debris and irrigant compared to closed chamber apparatus method.

## Figures and Tables

**Table 1:** Amount of debris and irrigant extruded apically in different study groups open and closed chambers.

Group	*n*	Debris (g)		Irrigant (mL)	
		Closed chamber (mean ± std. deviation)	Open chamber (mean ± std. deviation)	Closed chamber (mean ± std. deviation)	Open chamber (mean ± std. deviation)
NA	5	0.127 ± 0.007	0.206 ± 0.016	0.378 ± 0.044	0.952 ± 0.064
LS	5	0.151 ± 0.012	0.264 ± 0.016	0.518 ± 0.030	1.062 ± 0.051
CB	5	0.197 ± 0.012	0.298 ± 0.016	0.602 ± 0.059	1.156 ± 0.046
PU	5	0.481 ± 0.050	0.596 ± 0.013	0.708 ± 0.030	1.318 ± 0.028
EA	5	0.250 ± 0.015	0.335 ± 0.032	0.662 ± 0.029	1.204 ± 0.036
EV	5	0.011 ± 0.005	0.016 ± 0.005	0.088 ± 0.019	0.118 ± 0.038
Total	**30**	**0.203 ± 0.148**	**0.286 ± 0.176**	**0.493 ± 0.216**	**0.968 ± 0.406**

Group	*n*	Debris (g)		Irrigant (mL)	
		Closed	Open	Closed	Open

NA	5	0.127 ± 0.007	0.206 ± 0.016	0.378 ± 0.044	0.952 ± 0.064
WA	25	0.218 ± 0.159	0.302 ± 0.189	0.516 ± 0.044	0.972 ± 0.445

NA = no agitation; WA = with agitation.

**Table 2:** Comparison of extruded debris and irrigant between open and close chamber groups (Tukey HSD test).

SN	Comparison	“*P*” value of debris		“*P*” value of irrigant	
		Closed	Open	Closed	Open
1	NA versus LS	0.593	0.001	<0.001	0.009
2	NA versus CB	0.001	<0.001	<0.001	<0.001
3	NA versus PU	<0.001	<0.001	<0.001	<0.001
4	NA versus EA	<0.001	<0.001	<0.001	<0.001
5	NA versus EV	<0.001	<0.001	<0.001	<0.001
6	LS versus CB	0.039	0.074	0.019	0.033
7	LS versus PU	<0.001	<0.001	<0.001	<0.001
8	LS versus EA	<0.001	<0.001	<0.001	0.001
9	LS versus EV	<0.001	<0.001	<0.001	<0.001
10	CB versus PU	<0.001	<0.001	0.002	<0.001
11	CB versus EA	0.013	0.041	0.159	0.559
12	CB versus EV	<0.001	<0.001	<0.001	<0.001
13	PU versus EA	<0.001	<0.001	0.409	0.006
14	PU versus EV	<0.001	<0.001	<0.001	<0.001
15	EA versus EV	<0.001	<0.001	<0.001	<0.001
